# A New Grinding Force Model for Micro Grinding RB-SiC Ceramic with Grinding Wheel Topography as an Input

**DOI:** 10.3390/mi9080368

**Published:** 2018-07-26

**Authors:** Zhipeng Li, Feihu Zhang, Xichun Luo, Xiaoguang Guo, Yukui Cai, Wenlong Chang, Jining Sun

**Affiliations:** 1 School of Mechatronics Engineering, Harbin Institute of Technology, Harbin 150001, China; 13B908073@hit.edu.cn; 2 Centre for Precision Manufacturing, Design, Manufacture & Engineering Management, University of Strathclyde, Glasgow G1 1XJ, UK; yukui.cai@strath.ac.uk (Y.C.); wenlong.chang@strath.ac.uk (W.C.); 3 Key Laboratory for Precision and Non-Traditional Machining Technology, Dalian University of Technology, Dalian 116024, China; guoxg@dlut.edu.cn; 4 School of Engineering and Physical Science, Heriot Wat University, Edinburgh EH14 4AS, UK; jining.sun@hw.ac.uk

**Keywords:** grinding force model, rubbing, plastic, brittle fracture, protrusion height

## Abstract

The ability to predict the grinding force for hard and brittle materials is important to optimize and control the grinding process. However, it is a difficult task to establish a comprehensive grinding force model that takes into account the brittle fracture, grinding conditions, and random distribution of the grinding wheel topography. Therefore, this study developed a new grinding force model for micro-grinding of reaction-bonded silicon carbide (RB-SiC) ceramics. First, the grinding force components and grinding trajectory were analysed based on the critical depth of rubbing, ploughing, and brittle fracture. Afterwards, the corresponding individual grain force were established and the total grinding force was derived through incorporating the single grain force with dynamic cutting grains. Finally, a series of calibration and validation experiments were conducted to obtain the empirical coefficient and verify the accuracy of the model. It was found that ploughing and fracture were the dominate removal modes, which illustrate that the force components decomposed are correct. Furthermore, the values predicted according to the proposed model are consistent with the experimental data, with the average deviation of 6.793% and 8.926% for the normal and tangential force, respectively. This suggests that the proposed model is acceptable and can be used to simulate the grinding force for RB-SiC ceramics in practice.

## 1. Introduction

Reaction-bonded silicon carbide (RB-SiC) is a good candidate material for large space optical mirrors due to their high strength, high thermal conductivity, enhanced radiation stability, and thermal shock resistance characteristics [1,2,3]. To date, grinding with superhard fine abrasives is the primary method used in achieving the desired tolerances and surface integrity for engineering ceramic machining [4,5]. However, inherent high hardness and brittleness present a barrier to plastic removal of RB-SiC ceramics. During the grinding process, the interaction between abrasive grains and RB-SiC ceramics leads to unavoidable damage which consists of cracks, voids, dislocations, and stacking faults, etc. Those damages will affect the service life of the components, especially if the brittle fracture is the key factor. To minimize the damage induced by brittle fracture, several previous studies have been performed to evaluate the relationship between grinding force and removal behaviour [6,7]. Grinding force is a crucial indicating factor on the grinding quality, which means that whether cracks formed or not is directly controlled by the applied normal load in the grinding process. The form accuracy and ground device quality, especially surface and subsurface integrity, are strongly influenced by the grinding force. Therefore, the prediction and proposed controlling method of the grinding force is significant for improving the surface and subsurface integrity of ceramic components. Numerous researchers have attempted to model the force for surface grinding from theoretical and experimental approaches. Malkin et al. [8] argued that the grinding force should be decomposed into two parts, namely, cutting deformation and sliding force. Werner et al. [9] presented an empirical model by surperimposing all instantaneous frictional and cutting forces of individual edges in contact with the workpiece. However, Ge et al. [10] suggest that Werner’s model did not distinguish the sliding and cutting from the physical relationships in grinding, therefore, the author constructed a grinding force model which separated sliding, plowing, and cutting forces based on the analyses of the grinding trajectory and grain workpiece contact. Badger et al. [11] developed two methods for calculating the grinding force. One is based on Challen and Oxley’s 2D slip-line field model of the contact between grit and workpiece, another is based on Willams and Xie’s 3-D model of a three-dimensional asperity which generates a series of grooves on the workpiece. To gain accurate results, the grinding wheel profile and some material properties need to be measured. Afterwards, Hecker et al. [12,13] proposed a model for grinding force and power based on the probabilistic distribution of the undeformed chip thickness which was assumed to be distributed as a Rayleigh probability density function. However, most of above-mentioned models concerned grinding of metallic materials, which just involved rubbing, ploughing, and chip formation stages, whereas brittle fracture is the most significant distinction removal mechanism between ceramics and metallic materials. The adoption of them results in the prediction of hard and brittle materials having deviations. This indicates that the transition from ductile deformation to the brittle fracture removal mode must be considered when modelling the grinding force for ceramics. Therefore, Wu et al. [14] extended Hecker’s model and predicted the grinding force for brittle materials considering the co-existence of ductility of brittleness. In this model, the surface and subsurface damage was quantitative characterized, but the random distribution of grain height and size was not considered. Nevertheless, based on the random grit distribution, described by a stochastic grit density function, Chang and Wang [15] developed a stochastic grinding force. Cheng et al. [16] established a predictive grinding force model in micro-slot grinding of single-crystal sapphire. Even though different orientations of sapphire were taken into account, the brittle fracture physical characteristic was not exhibited in the model. Except for the above-mentioned studies, researchers also developed a novel grinding forces model of ultrasonic variation-assisted grinding for brittle materials, such as zirconia [17], alumina [18], and silica glass, and Al_2_O_3_ ceramic [19]. Most of them attempted to build a grinding force model according to the analysis of the motion trajectory of grits and material removal mechanisms. Despite many models that could be used to predict grinding forces, they require optimization and improvement. In particular, it can be determined that, considering the brittle fracture characteristic while, at the same time, combining the random distribution of grinding wheel grains, is the major impediment to modelling the grinding force for RB-SiC ceramics.

Consequently, in order to obtain a predictive model for ceramics, an improved theoretical force model was proposed in this paper, which takes the random distribution of abrasives, grinding trajectory, and different material deformation stages into consideration. The components of grinding force, contact length of the grinding wheel, and the workpiece were analysed first. Then, the corresponding critical depth of elastic, plastic, and brittle fracture stages were calculated. Afterwards, single-grain scratch rubbing, ploughing, and brittle fracture force were determined based on different interaction mechanisms. During the modelling process, the grain shape, protruding height of diamond grains, and random distribution of grinding wheel grain features were measured using an Alicona microscope and the obtained parameters were used as the input variables. Hence, the total grinding force was obtained by incorporating grains involved in each stage.

## 2. New Grinding Force Model

**Figure 1:**
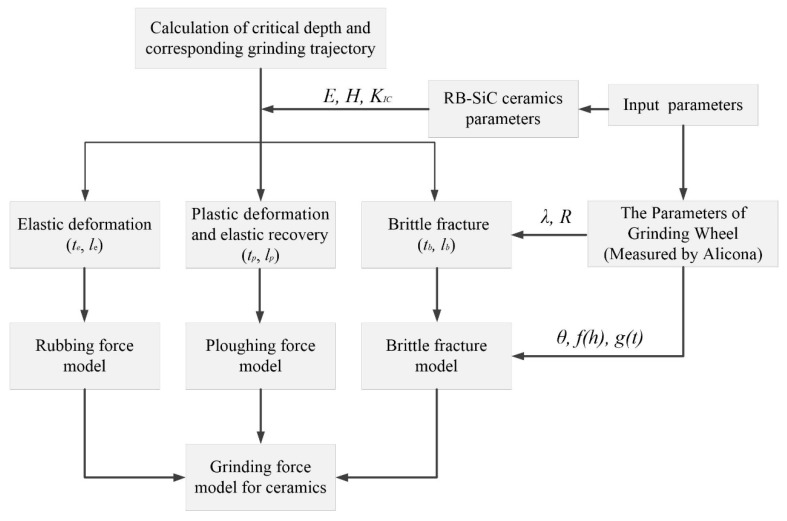
The diagram of the developing process of the proposed grinding force model.

The detailed structure of the developing process of the proposed grinding force model is illustrated in Figure 1. The distance between two continuous dynamic active grains, the effective radius of the indenter tip, and the RB-SiC ceramics’ physical properties were firstly taken as the input parameters to calculate the critical depth transition from elastic to plastic and, finally, to brittle fracture. Then, based on each stage of the critical depth, the total grinding force of RB-SiC ceramics was decomposed into different components and the corresponding stress state under a single grain at each stage was built. In the end, the amount of dynamic active grains participating in cutting, the protruding height of diamond grains, and the random distribution of grains were used to develop the total grinding force model. The novelties of the modelling approach lie in two aspects, i.e., developing the grinding force components, including rubbing, ploughing, and brittle fracture, separately, taking into consideration the brittle fracture removal mode which is particularly necessary for ceramics. Additionally, the random grinding wheel topography was chosen as an input parameter to compute the force.

During the grinding process, the grinding force is fully dependent on the grinding depth. On the basis of the grinding trajectory and material properties, the whole machining process during the interaction between grains and workpiece can be divided into four regimes, namely, elastic, plastic, chip formation, and brittle fracture. However, the inherent hardness and brittle characteristics of RB-SiC ceramics result in a slight space left for the ductile transition to brittle fracture (DTB). Therefore, the elastic deformation and elastic recovery at the rear of the indenter cannot be ignored in the modelling force, especially at the initial contact of nanoscale grinding. Additionally, the calculated minimum depth for chip formation is much larger than the depth for DTB (Section 2). It indicates that the ductile chip formation can be assumed not considered in the force modelling. This phenomenon can be explained by the large negative rake angle of diamond grains and material brittleness. That is to say, the fracture occurred in machining plays an important role in the material removal stage. As a result, in accordance with the critical depth of the elastic to plastic transition *t_e_*, and ductile to brittle transition *t_b_*, the material removal process can be divided into two parts as follows:

(1) $$\left\{ \begin{array}{l} \begin{array}{l} t<t_{e}(\mathrm{rubbing})\\ t_{e}<t<t_{b}(\mathrm{ploughing}) \end{array}\}\;(\mathrm{Ductile}\;\mathrm{region})\\ t>t_{b}(\mathrm{fracture})\;\;\}\;(\mathrm{Brittle}\;\mathrm{fracture}\;\mathrm{region}) \end{array}\right.\;$$

In terms of the depth of the gradient, the predictive model of grinding forces should be made of the rubbing force, ploughing force, and brittle fracture chipping force. The *F_T_* and *F_N_* forces can be expressed by Equations (2) and (3):

(2) $$F_{N}=F_{ne}+F_{np}+F_{nb}\;$$

(3) $$F_{T}=F_{te}+F_{tp}+F_{tb}\;$$

where *F_ne_*, *F_np_*, and *F_nb_* are the normal rubbing and ploughing force and fracture chip force, *F_te_*, *F_tp_*, and *F_tb_* are the tangential sliding and ploughing force and fracture chipping force.

### 2.1. Trajectory Length of a Single Diamond Grain Workpiece

**Figure 2:**
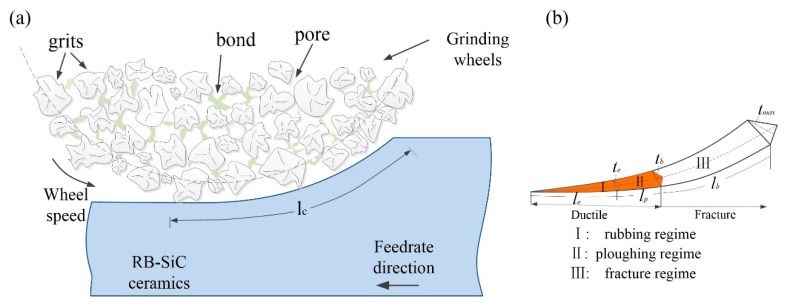
(**a**) The schematic diagram of grinding process; and (**b**) the three stages divided in a whole contact trajectory.

Based on a previous discussion of the different stages, the geometrical contact arc length between the workpiece and grinding wheel is indicated by:

(4) $$l_{t}=l_{e}+l_{p}+l_{b}\;$$

where *l_t_* is the ideal contact length equal to $l_{t}=\sqrt{ad_{s}}$, in which the motion and deformation of grinding wheels and workpiece are neglected, *a_p_* is the grinding depth, *d_s_* is the diameter of the grinding wheel, *l_e_*, *l_p_*, and *l_b_* are the contact length in elastic, plastic, and brittle fracture stages, respectively. As shown in Figure 2, from the proportional relationships it can be deduced:

(5) $$l_{e}=\frac{t_{e}}{t_{m}}l_{t}\;$$

(6) $$l_{p}=\frac{l_{t}}{t_{m}}(t_{b}-t_{e})\;$$

(7) $$l_{b}=l_{t}-l_{e}-l_{p}\;$$

where *t_m_* is the maximum undeformed chip thickness.

### 2.2. Dynamic Grinding Trajectory and Uncut Chip Thickness

#### 2.2.1. The Critical Depth for the Elastic to Plastic Transition

The parameters of the critical depth for each stage should be estimated first. For the elastic to plastic transition the maximum contact stress *P_max_* at the critical place can be obtained by [20]:

(8) $$P_{max}=\frac{2E_{r}a_{p}}{\pi R}\approx 1.6\frac{H}{2.8}\;$$

Then, the critical depth calculated based on Hertz theory is expressed by [21]:

(9) $$t_{e}=0.428a_{p}=\frac{P_{max}\pi R}{2E_{r}}=0.1223\frac{\pi HR}{E_{r}}\;$$

where *E_r_* is the composite elastic modulus, *a_p_* is the indentation depth induced by *P_max_*, and *R* is the effective radius of the indenter tip, which can be calculated by the following equation:

(10) $$E_{r}={\left(\frac{1-\upsilon_{1}^{2}}{E_{1}}+\frac{1-\upsilon_{2}^{2}}{E_{2}}\right)}^{-1}\;$$

where *E*_1_ and *E*_2_ is the elastic modulus of the workpiece and the diamond indenter, respectively. *υ*_1_ and *υ*_2_ are the Poisson’s ratio of the workpiece and diamond indenter, respectively.

#### 2.2.2. The Critical Depth of Cut for Chip Formation

The minimal depth of the cut for chip formation thickness *t_cr_* can be determined by the formula proposed by [22]:

(11) $$t_{cr}=R[1-\cos(\pi /4-\beta /2)]\;$$

where *β* is the friction angle that equal to arctan(*μ*), and *μ* is the apparent friction coefficient that can be get from our previous study [23].

#### 2.2.3. The Critical Depth for the Ductile to Brittle Transition

If it is assumed that the effect of the grinding parameters on material properties is ignored, the critical transition from ductile to brittle fracture can be determined by the material elastic modulus *E*_1_, hardness *H*_1_, and fracture toughness *K_IC_*. The depth of DTB can be predicted by the following equation [4]:

(12) $$t_{b}=\varepsilon (\frac{E_{1}}{H_{1}}){\left(\frac{K_{IC}}{H_{1}}\right)}^{2}\;$$

where *ε* is a constant as 0.15. Through the comparison, it can be found that the critical depth for chip formation (*t_cr_* = 147.43 nm) is much larger than the DTB (*t_b_* = 36.83 nm) depth. For this reason, the ductile chip formation force can be ignored in this model.

#### 2.2.4. The Maximum Undeformed Chip Thickness in Micro-Grinding

According to the grinding principle, for two continuous cutting grains the maximum underformed chip thickness *t_max_* can be expressed by [24]:

(13) $$t_{max}={\left(2\lambda \frac{v_{w}}{v_{s}}\sqrt{\frac{a}{d_{s}}}\right)}^{\frac{1}{2}}\;$$

where *λ* is the space between the dynamic active cutting grains, *d_s_* is the diameter of the grinding wheel, *v_w_* is the feed rate, and *v_s_* is the peripheral speed of the grinding wheel. From the kinematic trajectory and simplified considerations, it can be assumed that the active continuous cutting grains are at the same protrusion height. Thus, the space between the continuous cutting grains can be obtained from the profile of the grinding wheel topography, as depicted in Section 3.

### 2.3. Normal and Tangential Force per Single Grain

#### 2.3.1. Cutting Force in the Elastic Stage

**Figure 3:**
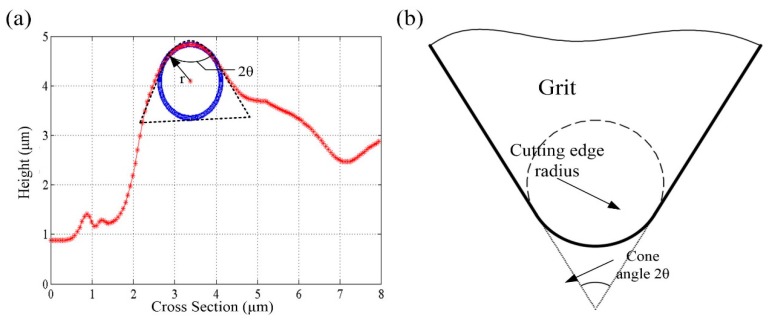
(**a**) Typical cross-sectional profile of grain; and (**b**) the simplified model of grain.

Figure 2a shows the schematic diagram of contact region between the grain and workpiece. The workpiece surface will undergo elastic deformation at the initial stage due to the small grinding depth. At such a depth, the grain tip can be regarded as a sphere contacting with the workpiece surface (illustrated in Figure 3). Based on Hertz theory [21], the normal force and tangential force can be derived from Equations (13) and (14):

(14) $$F_{ne}=\frac{4}{3}E_{r}R^{1/2}t^{3/2}\;$$

(15) $$F_{te}=\mu_{a}\frac{4}{3}E_{r}R^{1/2}t^{3/2}\;$$

where *μ_a_* is the adhesion fraction coefficient [23].

#### 2.3.2. Cutting Force in Plastic Stage

As the grinding depth increased, the workpice will start to deform plastically at the point where the yield criterion is satisfied, while the normal and tangential ploughing force can be obtained as follows:

(16) $$dF=\sigma_{y}dA\;$$

where *σ_y_* is the compressive yield stress at the contact area [25]:

(17) $$\sigma_{y}={\left(H^{4}/E\right)}^{1/3}\;$$

The contact projected area in the normal direction *A* and thrust direction *S* can be given by:

(18) $$A=\pi (2Rt-t^{2})/2\;$$

(19) $$S=R^{2}\cos^{-1}(\frac{R-t}{R})-(R-t)\sqrt{2Rt-t^{2}}\;$$

Thus, the normal and thrust force (plastic stage) can be obtained by submit Equations (17)–(19) into Equation (16):

(20) $$F_{np}=\frac{\pi \sigma_{y}(2Rt-t^{2})}{2}=\frac{\pi {\left(H^{4}/E\right)}^{1/3}(2Rt-t^{2})}{2}\;$$

(21) $$F_{tp}={\left(H^{4}/E\right)}^{1/3}(R^{2}\cos^{-1}(\frac{R-t}{R})-(R-t)\sqrt{2Rt-t^{2}})\;$$

#### 2.3.3. The Elastic Recovery Force at the Rear of the Tool in the Plastic Deformation Region

The grinding force caused by the elastic recovery of the material at the rear of the tool cannot be neglected. The spring-back height of the newly-machined surface can be estimated by [26]:

(22) $$t_{s}=\chi R\frac{H}{E}\;$$

where *χ* is a scaling constant for the best fit.

As the material is assumed to give a perfect elastic plastic response, the plastic depth results only in fthe plastic flow around the tip. Hence, the stress on the flank face is equal to *σ_y_*. The elastic deformation caused tangential force can be defined by:

(23) $$F_{en}=\sigma_{f}A_{e}=\chi \pi \sigma_{f}R^{2}H\frac{1}{E}(1-K_{2}\frac{H}{2E})\;$$

(24) $$F_{et}=\mu_{a}\chi \pi \sigma_{f}R^{2}H\frac{1}{E}(1-K_{2}\frac{H}{2E})\;$$

#### 2.3.4. Contact Force in the Brittle Zone

**Figure 4:**
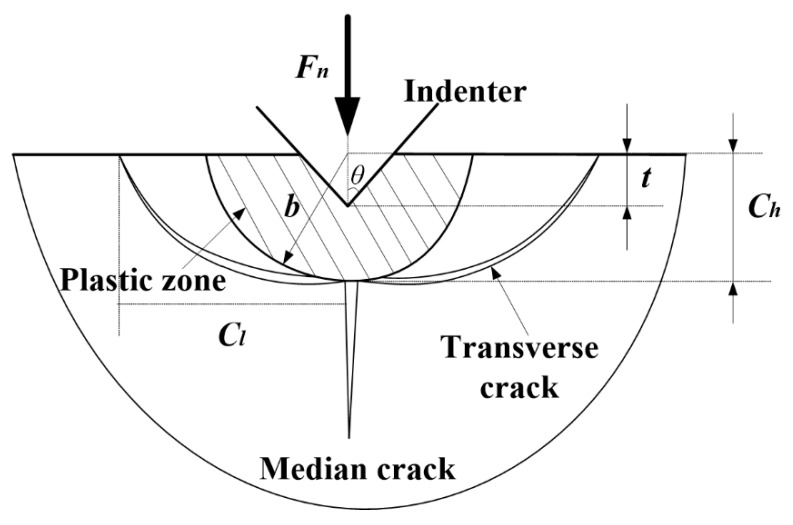
Illustration of the material removal volume in the brittle region during grinding.

While in the last stage *t* > *t_b_*, material is primarily removed in the brittle fracture mode. In this regime, the generation and propagation of cracks are the main reason of facture chipping. As shown in Figure 4, median cracks will form and propagate first beneath the grits with the increase of the normal load. At the following unloading process, the lateral crack will be generation due to the mismatch of the residual stress between the interface of elastic and plastic zone. Afterwards, the chipping will generate and the materials will be removed. The depth *C_h_* and length *C_l_* of the lateral crack can be obtained by the following equations [27]:

(25) $$C_{l}=C_{2}{\left(\frac{1}{\tan\theta }\right)}^{5/12}{\left[\frac{E^{3/4}}{HK_{IC}{\left(1-v^{2}\right)}^{1/2}}\right]}^{1/2}{\left(F_{nb}\right)}^{5/8}\;$$

(26) $$C_{h}=C_{2}{\left(\frac{1}{\tan\theta }\right)}^{1/3}\frac{E^{1/2}}{H}{\left(F_{nb}\right)}^{1/2}\;$$

where *θ* is the half apex angle of the indenter, *C*_2_ is a dimensionless constant, which is independent of material-indenter system, and *C*_2_ = 0.226 [23].

Moreover, the plastic deformation zone depicted in Figure 4 is approximated by a semicircle of radius *b* [28]. The plastic zone radius is expressed as a function of not only the load and material properties, but also of the grit’s geometry. An empirical relationship between the yield strength *σ_y_* and elastic modulus, the Poisson ratio is used to obtain the plastic zone radius:

(27) $$b={\left(\frac{3\left(1-2\nu \right)}{5-4\nu }+\frac{2\sqrt{3}}{\pi \left(5-4\nu \right)}\frac{E}{\sigma_{y}}\cot\theta \right)}^{\frac{1}{2}}a\;$$

where a=ttanθ. As described above, the lateral cracks initiated at the bottom of plastic deformation zone, therefore, the depth *b* can be assumed to be equal to *C_h_*. Thus, combining Equations (23) and (24), the final force in brittle fracture regime can be written as:

(28) $$F_{nb}=Ct^{2}H^{2}{\left(\tan\theta \right)}^{8/3}(\frac{3\left(1-2\upsilon \right)}{E(5-4\upsilon )}+\frac{2\sqrt{3}}{\pi \left(5-4\upsilon \right)}\frac{1}{\sigma_{y}}\cot\theta )\;$$

(29) $$F_{tb}=Ct^{2}H^{2}{\left(\tan\theta \right)}^{8/3}\frac{C_{l}}{C_{h}}(\frac{3\left(1-2\upsilon \right)}{E(5-4\upsilon )}+\frac{2\sqrt{3}}{\pi \left(5-4\upsilon \right)}\frac{1}{\sigma_{y}}\cot\theta )\;$$

where *C* = *1/C*_2_. Thus, *F_nb_* and *F_tb_* can be calculated from the above Equations (28) and (29), respectively.

### 2.4. Measurement of the Grinding Wheel via an Alicona Microscope

#### 2.4.1. The Topography of the Grinding Wheel Surface

**Figure 5:**
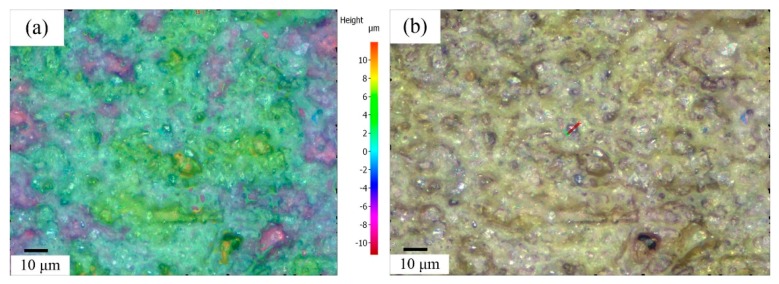
The topography of a #6000 grinding wheel. (**a**) Three Dimension; (**b**) Two Dimensional.

To characterize the cutting area surface, the 3D topography data of grinding wheel was directly measured by an Alicona microscope. The surface digitization is based on focus-variation. The resolution of minimum vertical repeatability is less than 0.12 nm. The data coexistence of longitudinal, lateral, and height of the wheel topography are necessary to identify the diamond grains’ distribution and dimensions. Figure 5 shows the topography of the #6000 resin bond and 100% grain density grinding wheel which was measured using a 50× objective.

#### 2.4.2. The Parameters of the Cutting Edge Radius and Cone Angle

**Figure 6:**
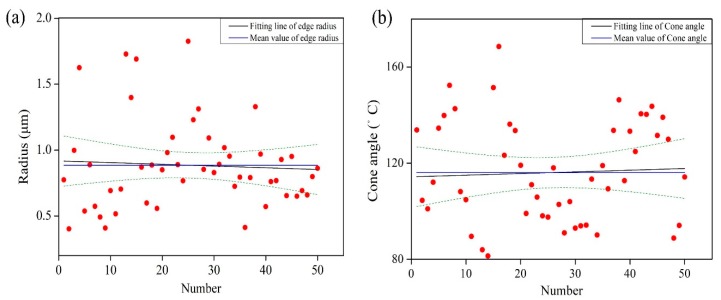
The measured data of grains radius (**a**); and the cutting cone angle (**b**).

Each digitized image is processed to extract the wheel surface information in the context of average cutting edge diameter, average cutting edge angle, average space between the dynamic active grains, and corresponding static density as a function of the radial distance into the wheel. Figure 3a shows the typical cross-section profile of single grain which chosen from Figure 5b. As shown in Figure 3b, the grain can be simplified as a cone shape with sphere tip. The dimension of the tips was fitted using MATLAB software (MathWorks, Pallini, Greece) with the method of least squares. Figure 6 shows the averaged value of cutting edge radius and cone angle that obtained by analysing a population of grains.

#### 2.4.3. Determination of Active Grains Protrusion Height and Number in Each Stages

**Figure 7:**
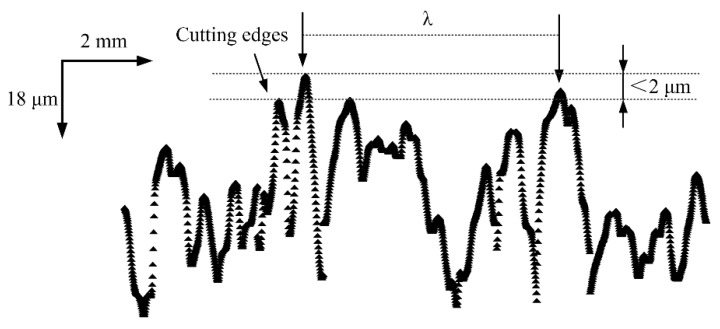
The traced profile along the periphery of grinding wheel.

Figure 7 shows the cross sectional profile along the periphery of grinding wheel. Owing to the interaction between grains, not all of the grains would participate in the cutting stage. Malkin [29] proposed that the grains and cutting edges can be evaluated through setting the threshold value. For simplify considerations, it will be assumed that the active continuous cutting grains are at the same protrusion height as depicted in Figure 7.

**Figure 8:**
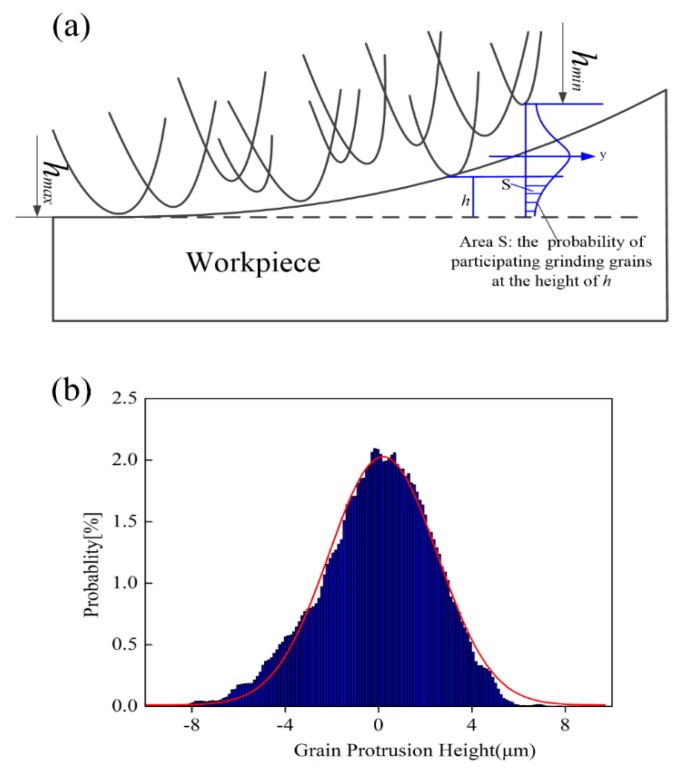
(**a**) Schematic of the grain size distribution showing maximum and minimum protrusion height of the grain and the probability distribution of other sizes of the grains; and (**b**) normal distribution plot of the frequency verse the grain protrusion height.

Figure 8 illustrates the details of grain protrusion height on the grinding wheel surface, which distribute in the form of normal distribution. The Gaussian function was applied to fit the data as follows:

(30) $$f(h)=\frac{1}{\sqrt{2\pi }\cdot \sigma }e^{-\frac{{\left(h-\delta \right)}^{2}}{2\sigma^{2}}}\;$$

where *σ* is the standard deviation and *δ* is the mean value of grain protrusion height.

The active grains number in each segment can be determined by:

(31) $$\Delta N=N_{a}\cdot l_{total}\cdot w\cdot \int_{h_{max}}^{\Delta h}f(h)dh\;$$

where *N_a_* is the average number of grains per units area, *h_max_* is the value of highest grain protrusion, *w* is the contact width of grinding and Δh is the difference value between *h_max_* and the corresponding height in each stages.

Additionally, *N_a_* can be approximately estimated from [30]:

(32) $$N_{a}=\frac{100}{d^{2}}\times {\left(\varphi (D)\right)}^{\frac{2}{3}}\;$$

where *d* and φ(D) are the average diameter and volume fraction of diamond grain.

### 2.5. Superposition of Single Grain Grinding Forces

As shown in Figure 8b the cutting depth has relationship with grain protrusion height, the cutting depth probability density can be describe as:

(33) $$g(t)=-\frac{1}{\sqrt{2\pi }\sigma }e^{-\frac{{\left(h_{\max}-t-\delta \right)}^{2}}{2\sigma^{2}}}\;(0<t<10\;\mu m)\;$$

By integrating of the tangential and normal force model per grain in different stage, the total tangential and normal force at each stage can be expressed as:

First stage, $0<t<t_{e}$

(34) $$F_{n}=\Delta N\cdot E(F_{ne})=\Delta N\int_{0}^{t_{m}}F_{ne}g(t)dt=N_{a}l_{t}w\int_{h_{m}-t_{m}}^{h_{m}}\int_{0}^{t_{m}}F_{ne}g(t)f(h)dtdh\;$$

(35) $$F_{t}=\Delta N\cdot E(F_{te})=\Delta N\int_{0}^{t_{m}}F_{te}g(t)dt=N_{a}l_{t}w\int_{h_{m}-t_{m}}^{h_{m}}\int_{0}^{t_{m}}F_{te}g(t)f(h)dtdh\;$$

Second stage, $t_{e}<t<t_{b}$

(36) $$\begin{array}{l} F_{n}=\Delta N_{1}\cdot E(F_{ne})+\Delta N_{2}\cdot E(F_{np})+\Delta N_{2}\cdot E(F_{en})\\ =N_{a}l_{t}w(\int_{h_{m}-(t_{m}-t_{e})}^{h_{m}}\int_{0}^{t_{m}-t_{e}}F_{np}g(t)f(h)dtdh+\int_{h_{m}-t_{m}}^{h_{m}-(t_{m}-t_{e})}\int_{t_{m}-t_{e}}^{t_{m}}F_{ne}g(t)f(h)dtdh+F_{en}\int_{h_{m}-(t_{m}-t_{e})}^{h_{m}}f(h)dh) \end{array}\;$$

(37) $$\begin{array}{l} F_{t}=\Delta N_{1}\cdot E(F_{te})+\Delta N_{2}\cdot E(F_{tp})+\Delta N_{2}\cdot E(F_{et})\\ =N_{a}l_{t}w(\int_{h_{m}-(t_{m}-t_{e})}^{h_{m}}\int_{0}^{t_{m}-t_{e}}F_{tp}g(t)f(h)dtdh+\int_{h_{m}-t_{m}}^{h_{m}-(t_{m}-t_{e})}\int_{t_{m}-t_{e}}^{t_{m}}F_{te}g(t)f(h)dtdh+F_{et}\int_{h_{m}-(t_{m}-t_{e})}^{h_{m}}f(h)dh) \end{array}\;$$

The third stage, $t>t_{b}$

(38) $$\begin{array}{l} F_{n}=\Delta N_{1}\cdot E(F_{nb})+\Delta N_{2}\cdot E(F_{np})+\Delta N_{3}\cdot E(F_{ne})\\ =N_{a}l_{t}w(\int_{h_{m}-(t_{m}-t_{b})}^{h_{m}}\int_{0}^{t_{m}-t_{b}}F_{nb}g(t)f(h)dtdh+\int_{h_{m}-(t_{m}-t_{e})}^{h_{m}-(t_{m}-t_{b})}\int_{t_{m}-t_{b}}^{t_{m}-t_{e}}F_{np}g(t)f(h)dtdh+\int_{h_{m}-t_{m}}^{h_{m}-(t_{m}-t_{e})}\int_{t_{m}-t_{e}}^{t_{m}}F_{ne}g(t)f(h)dtdh\\ +F_{en}\int_{h_{m}-(t_{m}-t_{e})}^{h_{m}-(t_{m}-t_{b})}f(h)dh)\; \end{array}\;$$

(39) $$\begin{array}{l} F_{t}=\Delta N_{1}\cdot E(F_{tb})+\Delta N_{2}\cdot E(F_{ts})+\Delta N_{3}\cdot E(F_{te})\\ =N_{a}l_{t}w(\int_{h_{m}-(t_{m}-t_{b})}^{h_{m}}\int_{0}^{t_{m}-t_{b}}F_{tb}g(t)f(h)dtdh+\int_{h_{m}-(t_{m}-t_{e})}^{h_{m}-(t_{m}-t_{b})}\int_{t_{m}-t_{b}}^{t_{m}-t_{e}}F_{tp}g(t)f(h)dtdh+\int_{h_{m}-t_{m}}^{h_{m}-(t_{m}-t_{e})}\int_{t_{m}-t_{e}}^{t_{m}}F_{te}g(t)f(h)dtdh\\ +F_{et}\int_{h_{m}-(t_{m}-t_{e})}^{h_{m}-(t_{m}-t_{b})}f(h)dh)\; \end{array}\;$$

However, the force model developed above is based on theoretical analyse, which neglect the effects of grinding thermal, cutting depth error caused by stiffness of the machine, and imperfect grain geometry. Therefore, three empirical constant *K*_1_, *K*_2_, *K*_3_ should be added to modify the force error produced in rubbing, plastic, and brittle fracture stages.

## 3. Experimental Setup and Procedure for Model Validation

**Figure 9:**
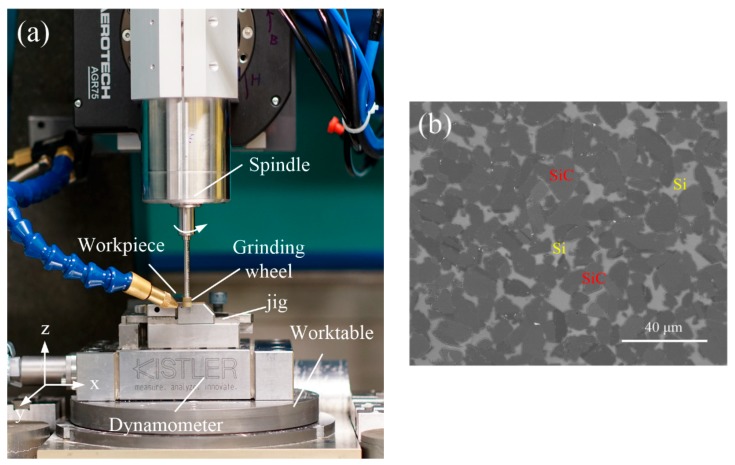
Experiment setup used to validate the proposed model of grinding forces (**a**) and (**b**) SEM image of surface morphology of the polished specimen.

To determine the experimental coefficients and verify the force prediction model presented in this work, grinding experiments were carried out on a hybrid ultra-precision micromachine (micro-3D) under dry cutting. The experiment set up is shown in Figure 9a. During grinding process, the grinding forces are measured by a three-component piezoelectric dynamometer Kistler 9129 AA. Each set of grinding parameters was repeated thrice and the average of three measured value was taken as the final results.

The material tested in present study is RB-SiC ceramics (Goodfellow Cambridge Ltd., Huntingdon, UK), which mainly consist of 90% of SiC phase with diameter of 10 μm and nearly 10% of Si phase (as shown Figure 9b). Table 1 listed some typical material properties of RB-SiC ceramics. The workpieces with dimensions of 12.5 × 12.5 × 5 mm are clamped on the worktable (Figure 9a). A resin bonded diamond grinding wheel with mesh number of #6000 (grit size of 15 μm), diamond concentration of 100%, diameter 6 mm, and width 8 mm was used. The grinding wheel was trued using an oilstone stick. The grinding wheel truing conditions are under a wheel speed of 2 m/s, the depth of cut 2 μm, and the transverse feed rate of 0.5 mm/s. In the tests, the grinding speed, feed rate and depth of cut were considered as machining parameters. Experimental parameters for determining coefficient and model calibration, verifying model are given in Table 2 and Table 3, respectively. To study the material removal characteristics and the influence of the RB-SiC microstructures, the machined surface topography was measured by an SEM (dual beam FEI Helios Nanolab 600i, Thermo Fisher Scientific, Waltham, MA, USA).

## 4. Results and Discussion

### 4.1. The Topography of Grinding RB-SiC

**Figure 10:**
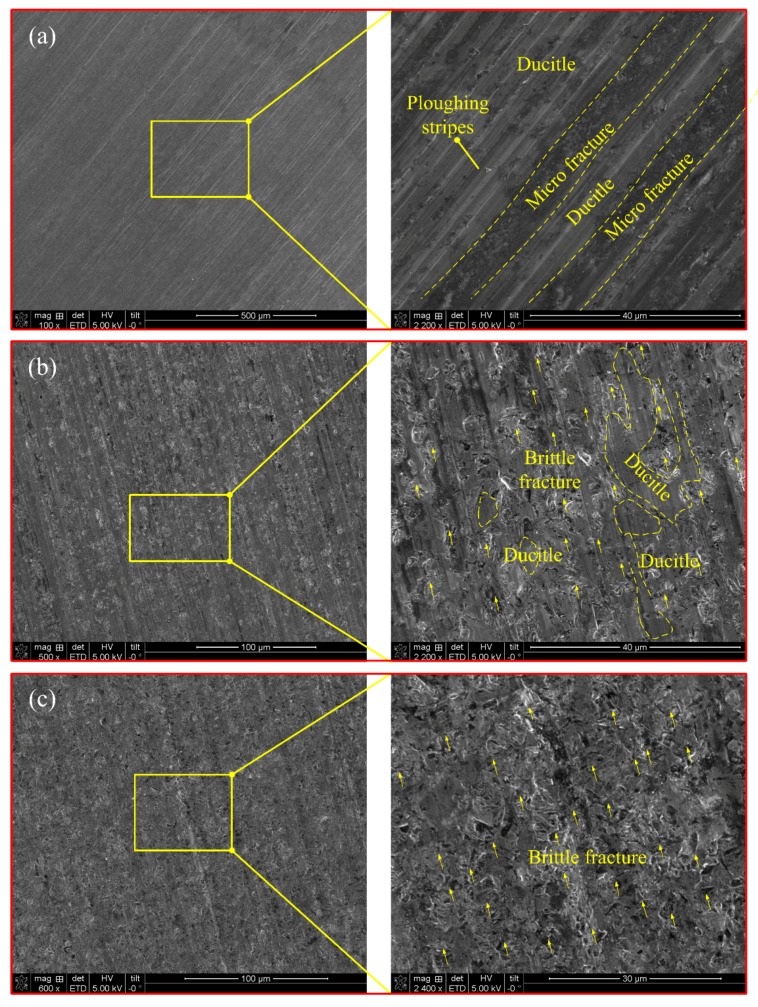
Comparison of the grinding surface morphology with increased *t_max_* (**a**) *n_s_* = 6000 r/min, *a_e_* = 2 μm, *v_w_* = 1 mm/s; (**b**) *n_s_* = 15,000 r/min, *a_e_* = 10 μm, *v_w_* = 8 mm/s; and (**c**) *n_s_* = 20,000 r/min, *a_e_* = 20 μm, *v_w_* = 12 mm/s.

Surface topography is one of the most important requirements in many engineering applications, as it is considered an important index of product quality. Figure 10 shows the typical SEM micrographs of ground surface morphology which obtained with the increase of grinding *t_max_*. It can be observed that three typical areas: (1) micro-fracture area; (2) ductile area (induced by ploughing stripes); and (3) macro-fracture area are generated on the machined topography. However, there are no obvious ductile debris particles appeared. Besides, the surface integrity obtained with relative small *t_max_* (Figure 10a) appears to be better than that shown in Figure 10b,c. This illustrated that brittle fracture become the primary removal mode gradually with the increase of *t_max_*. Therefore, it is reasonable to believe that the material removal stages that divided in Section 2 is suitable.

### 4.2. Determination of Experimental Coefficients

**Figure 11:**
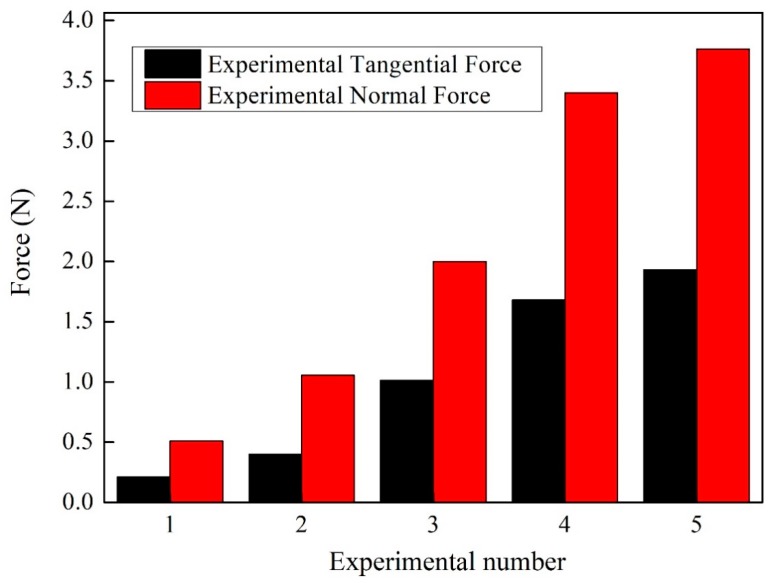
The experimental results of normal and tangential forces used for calibration.

The grinding force can be measured through experiments, then the value of unknown empirical constant *K*_1_, *K*_2_, *K*_3_, and *χ* can be determined through the least square estimation method. Table 2 list the machining parameters of five group experiments for calibration of the force model. To minimize errors induced by random wheel topography, three runs of each calculation are performed and mean values are illustrated in Figure 11. Through the calculation, the parameters are of *K*_1_, *K*_2_, *K*_3_, and *χ* are equal to 0.1228, 8.9934, 0.4116, and 0.1282 respectively. Then, combining the coefficient with Equations (34)–(39), the complete theoretical force model can be used to predict the grinding force in practice.

### 4.3. Force Model Calibration and Verification

**Figure 12:**
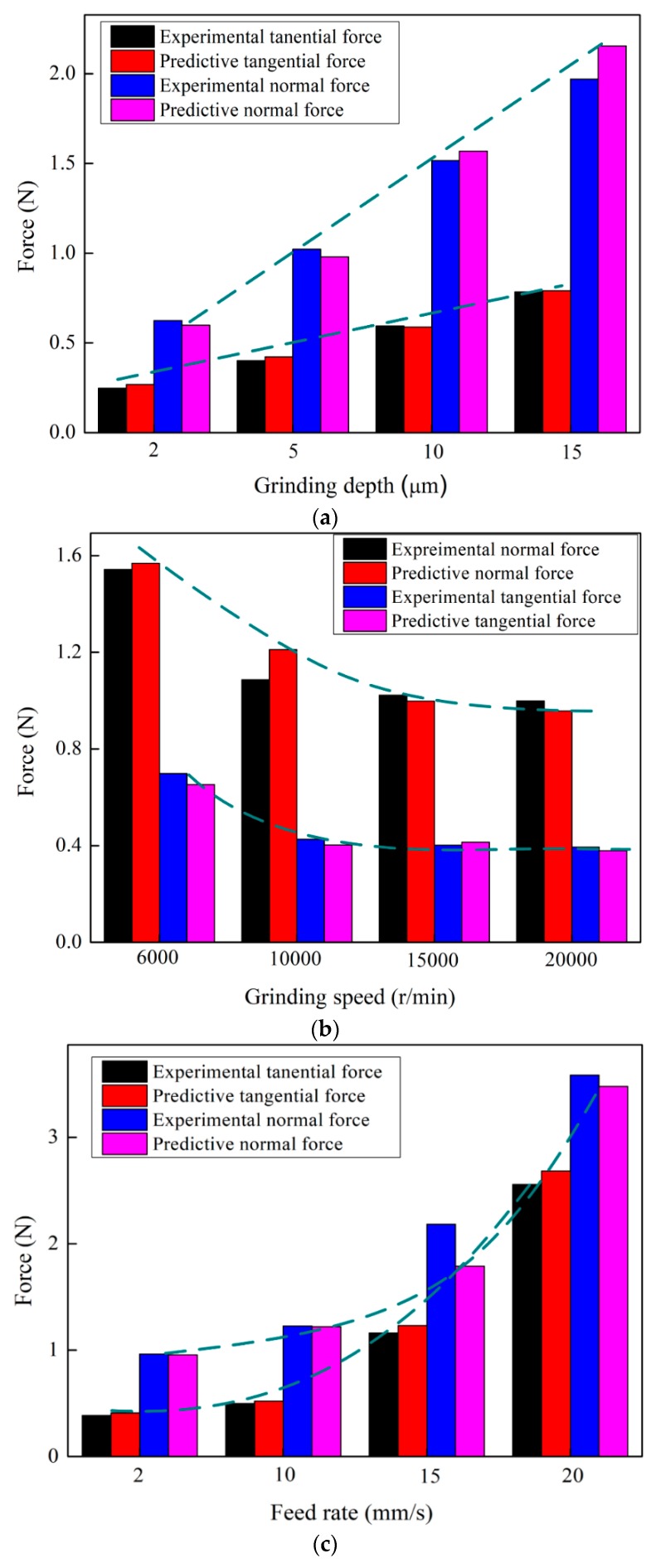
The experimental results for assessing the accuracy of the proposed model. (**a**) Grinding depth vs. Force; (**b**) Grinding speed vs. Force; (**c**) Feed rate vs. Force.

To validate the grinding force model proposed in this paper, another 12 groups of experiments with varied grinding depth, grinding speed and feed rate were performed. The machining parameters for verifying the model are shown in Table 3 The predictions of tangential and normal micro-grinding force to RB-SiC ceramics are calculated using the proposed models Equations (38) and (39). The comparison results for normal and tangential forces are presented in Figure 12. It could be find that the prediction values are consistent with the experimental results. The average percentage of the deviation in normal force and tangential force are 6.793% and 8.926%, respectively. Meanwhile, it can be seen that as grinding depth increased, the tangential and normal grinding force increased with linear relationship. However, both of tangential and normal force decreased with the increase of grinding wheel speed due to the ∆*N* and the corresponding *t_max_* change slightly. The grinding wheel speed will result in reverse effect on the maximum cutting depth. Therefore, the grinding force shows a downward trend with the increasing grinding wheel speed. Additionally, it should be note that within the chosen parameter of feed rate, the grinding force exhibits a significant upward trend and non-linear proportional to feed rate. In this process, the increased *t_max_* lead to more material removal volume in the brittle region and the corresponding brittle grinding force increased intensely.

## 5. Conclusions

A theoretical grinding force model for RB-SiC ceramics has been established with the consideration of rubbing, plastic flow, and brittle fracture removal mechanisms. Additionally, the parameters of the grains’ random distribution and protrusion was measured with the aid of an Alicona microscope were fed back into the model to integrate the individual grain force. Accurate calibration experiments were conducted to obtain empirical coefficients under different grinding parameters. The validity of the model is proved by comparing the experimental data with the predicted values.

(1) The grinding wheel topography measurement results suggest that the height of grain protrusion distribution obeys the normal distribution law.
(2) The SEM observations of grinding surface topography indicated that ploughing and brittle fracture were the dominate deformation mechanism. Meanwhile, no ductile chips were found within the chosen grinding parameters. These phenomena revealed that the assumed grinding force components including rubbing, ploughing, and brittle fracture is feasible.
(3) The feed rate has the most significant impacts on the grinding force, and the grinding force is proportional to the feed rate and grinding depth. In contrast, increasing the grinding wheel speeds will result in a downward trend in the grinding force.
(4) The validation experimental results show that the predicted grinding force model can be employed to simulate the grinding forces. The average percentage of the deviation of the normal force and tangential force are 6.793% and 8.926%, respectively. Therefore, the proposed methodology was proven to be able to capture the actual grinding process of ceramics.

## Figures and Tables

**Table 1:** Workpiece material properties.

Workpiece	RB-SiC
Elastic modulus (Gpa)	390
Vickers hardness (Kgf·mm^−2^)	3000
Compressive strength (Mpa)	2000
Fracture toughness KIC (Mpa·m^1/2^)	4.0
Thermal Expansion Coefficient (×10^−6^/°C)	3
Thermal Shock Resistance (°C)	400
Density ρ (g/cm^3^)	3.1

**Table 2:** Grinding parameters for determining coefficient.

Exp. No.	Grinding Depth *a_e_* (μm)	Grinding Speed *n_s_* (m/s)	Feed Rate *v_w_* (mm/s)
1	2	6000	1
2	5	10,000	5
3	10	15,000	8
4	15	20,000	10
5	15	20,000	12

**Table 3:** Model calibration between predictive and experiment results.

Exp. No.	Grinding Depth *a_e_* (μm)	Grinding Speed *v_s_* (m/s)	Feed Rate *v_w_* (mm/s)
1	2		
2	5		
3	10	6000	2
4	15		
5		6000	
6	5	10,000	5
7		15,000	
8		20,000	
9			2
10	5	15,000	10
11			15
12			20
